# Photodiagnosis and photodynamic recognition of cervical cancer with SEM and AFM images

**DOI:** 10.1371/journal.pone.0316544

**Published:** 2025-02-06

**Authors:** Sevcan AYTAÇ, Gürkan ÖZBEY

**Affiliations:** 1 Department of Electronic Technology, Firat University, Elazığ, Turkey; 2 Department of Obstetrics and Gynecology, Private Anadolu Hosital, Elazığ, Turkey; Purdue University, UNITED STATES OF AMERICA

## Abstract

So far, the number of patients who die from cancer is quite high. Continuation of early detection research is important to reduce the number of deaths due to cancer. At the time of the literature review, images of the same patients taken from Scanning Electron Microscope (SEM) and Atomic Force Microscope (AFM) for early diagnosis of cervix cancer have not been addressed to date. This article, Photodiagnosis and Photodynamics with SEM and AFM images are valuable in recognizing cervical cancer and starting treatment early. Simultaneous examination of the, Photodiagnosis and Photodynamics with SEM and AFM cervix images of patients will provide us with a far more powerful solution than a one-way solution. Daubechies (db2, db3, db4, and db5), Coiflet (coif5, coif4, coif3, and coif2), Symlet (sym5, sym4, sym3, and sym2), and Biorthogonal (bior1.3, bior2.8, bior1.5, and bior3.3) 16 discrete wavelet transformation families (DWTF) have been applied to AFM and SEM images. One approximate and three detail coefficients have been obtained for each one AFM and SEM cervix images. Homogeneity, contrast, angular second moment, entropy, mean, standard deviation, correlation, cluster prominence, dissimilarity, and cluster shade values have been calculated for each of these one approximate and three detail coefficients. The classification rate found by the averages of the results obtained from the DWTF_JSD, DWTF_HD and DWTF_TD algorithms for AFM and SEM cervix images are 98.29% and 97.10%, respectively. According to these results, it has been determined that SEM images have lower classification rate than AFM images. It has been also observed that the surface roughness of the mAFM images was larger than nAFM and bAFM images. But, it was observed that the volume of particles of the mAFM images has been smaller than nAFM and bAFM images.

## 1. Introduction

Scanning Electron Microscopy (SEM) [1] was invented for the first time in 1935 by a researcher, Knoll, to study the surfaces of solid bodies [2]. Examination of the surface characteristics of cells with SEM reveals the findings that can not be obtained with light microscopy and transmission electron microscopy [2, 3]. Obtaining the image with a SEM microscope is the result of evaluation of the incoming electron beams after reflection from the sample surface [2, 4]. AFM is a type of scanning tip microscope. The AFM developed by Heinrich Rohrer and Gerd Binnig about 20 years ago, works by detecting a sample surface of a mechanical tip. The device is a very high resolution scanning force microscope, but it also involves investigating the mechanical properties of the surfaces, even of nanometer scale samples. The most important advantage is that the samples can be viewed directly in any environment without special preparation. Additionally, nanobiomechanical quantities such as surface roughness and particle volume can be measured with AFM. Surface roughness, often abbreviated as roughness, is a component of surface texture. It is measured by the deviations in the direction of the normal vector from the ideal form of a real surface. If these deviations are large, the surface is rough; If they are small, the surface is smooth. Its unit is (nanometer (*nm*)). Also, unit of particle volume is (micrometer (*μm*)). A micrometer is one thousandth of a millimeter and one millionth of a meter [2, 5, 6].

Nowadays, it has become a very important issue the extraction of the effective features for texture-based and content-based images [7]. Nowadays, the analysis of tissue-type images is used in many fields such as medical imaging, industrial research, document segmentation and classification, recognition and classification [8]. Gaussian Markov Random Fields (GMRF) which be probabilistic models and autoregulation methods has been investigated for the analysis of another study [8, 9]. Various methods have been developed for the analysis of tissue type images. Statistical methods are one of these [10, 11]. Analysis of texture-type images have recently shifted to multi-resolution time-frequency methods such as Gabor filters [12] and wavelet decomposition [13–15]. These methods provide very good possibilities for analyzing and classifying tissue type images [13–15]. In order to fully analyze, categorize and recognize the tissue type images, the distinguishing features to be extracted from these images must be sufficient. We can classify the studies that used old feature extraction methods used for analysis of texture-like images to be as follows:

Studies using statistical features of first and second order of tissue type images [16, 17]. Studies using Gibbs Random Fields and GMRF are in references [18–20]. Studies recommending of local linear transformations are in references [21, 22]. These classical methods must largely show the same characteristics of different regions of tissue type sections. However, some of the restrictions given above still cannot ensure this. However, the biggest problem encountered in texture analysis by the above-mentioned classical methods is that the same or similar characteristics can not be obtained from each of the randomly selected images of the same size in the image. Therefore, in this article wavelet transformation method, which has become one of the most popular methods in tissue type image analysis, is used. Because wavelet transform offers the convenience of expressing that these groups of pixels are the same pieces of image by introducing robust features that can minimize the characteristic difference between different regions of the same size selected randomly [23]. Furthermore, the wavelet transform improves the robust recognition performance of the images in different metrics with the multi-resolution feature it brings. One of the biggest factors is that the high and low bands used for DWT can remain the identical for a fraction of different consecutive scales [23]. The main objectives of this study can be summarized as follows:

- To demonstrate the performance of the DWTF method which combined with Homogeneity, contrast, angular second moment, entropy, mean, standard deviation, correlation, cluster prominence, dissimilarity, and cluster shade properties of cervical AFM and SEM cells.
- To identify cervical AFM and SEM cells using wavelet transform based JSD, HD, and TD algorithms in the analysis of cervical images.

In this article, normal SEM cells are nSEM. Benign SEM cells are bSEM. Malign SEM cells are mSEM. Also, normal AFM cells are nAFM. Benign AFM cells are bAFM., and malign AFM cells are mAFM. SEM cells are acquired from Sciece Faculty SEM Laboratory in the Fırat University. Besides, AFM cells are acquired from Nanotechnology Laboratory in the Fırat University. In this article a total of 45 images of each the cervix cells (nSEM, bSEM, mSEM nAFM, bAFM, and mAFM) type were used in 512 x 512 sizes. Every of these cervical cells was described into regions, which consists 4 pieces of nonintersecting 256x256 cervical cells. Therefore, 3x180 quantities were occurred from cervical cells which have 3x4x45 quantities. So, total 540 SEM cells have been taken as 3x4x45 (nSEM, bSEM, and mSEM). Also, total 540 AFM cells have been taken as 3x4x45 (nAFM, bAFM, and mAFM). The 270 pieces of these 540 and the remaining 270 pieces of them for both SEM cells and for AFM cells were used for training and testing steps of suggested algorithm in this study separately. For this purpose, 16 DWTFVs are applied to each of the 256 × 256 image regions obtained for each of the 45 images. Then, one approximate and three detail coefficients has been calculated with DWT method according to level 3 for each of the 256 × 256 size images. Homogeneity, contrast, angular second moment, entropy, mean, standard deviation, correlation, cluster prominence, dissimilarity, and cluster shade features have been calculated for each of the these approximate and detail coefficients. Thus, DWTFVs in the 4x10 size have been obtained for each 256x256 image. Homogeneity, contrast, angular second moment, entropy, mean, standard deviation, correlation, cluster prominence, dissimilarity, and cluster shade feature values were given for training to the DWTF_JSD, DWTF_HD and DWTF_TD algorithms.

This article can be summarized as follows: In Chapter 1 has been discusses from previous work. In Section 2 has been given general information’s in about the DWTF, DWTF_JSD, DWTF_HD and DWTF_TD algorithms. The recommended system steps in section 3 are described. Experimental study results of categorization of cervical images in section 4 are given. The results obtained in Section 5 are discussed.

## 2. Material and methods

The study adhered to the Declaration of Helsinki and was approved by the local ethics review board. In this study, we used real medical images in order to evaluate the cervical cancer diagnosis. We sought Non-Interventional Clinical Researches Ethics Board in Firat University in order to obtain patient records coming from Elazig and its neighbor cities in Turkey. The medical image usage was approved with decision number of 07–05 by the related board. After this, we collected the patients’ medical records belonging to Firat University Hospital. Patients’ medical records were taken suitably to the ethics rules of the hospital. Medical tissue samples are determined from Firat University Medicine Faculty Pathology Laboratory. Our study is a retrospective study. Ethical approval was obtained before starting the study. It was conducted in accordance with the Declaration of Helsinki. Our relevant ethics committee approval has been uploaded to the other files sections. Our study started on 01/05/2016 and ended on 01/05/2021. Our study is a retrospective study. The data used in the study were obtained from medical archive records after ethical approval. Our ethics committee therefore did not require a consent form and waived the need for consent. Therefore, consent was not obtained. No patient data under the age of 18 was used in the medical data used in the study. The medical records required for our study were accessed between 01/05/2016 and 01/05/2021. All data were obtained from archived medical records after obtaining relevant permissions and approvals. No extra intervention was given to the participants.

### 2.1. Discrete Wavelet Transform Families (DWTF)

Biorthogonal (bior), Symlet (sym), Coiflet (coif), and Daubechies (db) discrete wavelet transformation families have been applied to AFM and SEM images. The db2, db3, db4, and db5 which be types of the db wavelet family have been applied to the AFM and SEM images. The coif2, coif3, coif4, and coif5 which be types of the Coif wavelet family have been applied to the AFM and SEM images. The sym2, sym3, sym4, and sym5 which be types of the sym wavelet family have been applied to the AFM and SEM images. The bior1.3, bior2.8, bior1.5, and bior3.3 which be types of the bior wavelet family have been applied to the AFM and SEM images.

#### 2.1.1. Daubechies wavelet

Daubechies is the orthogonal property of the wavelike family and has asymmetrical properties that cause large phase distortions. Therefore, the Daubechies wavelet family should not be used in applications where information must be kept [24].

#### 2.1.2. Coiflet wavelet

Coiflet wavelet family is steep, and shows a close symmetry feature. This close symmetry feature ensures that the Coiflet ripple is of near linear phase [24].

#### 2.1.3. Symlet wavelet

Symlet wavelet is vertical, and is in feature of close symmetric. This feature means low information deterioration. At Nth grade, a Symlet wave has N number of collapse torques for a support width of 2N-1. Symlet wavelets are very similar to Daubechies wavelets, except for its very symmetrical feature [24].

#### 2.1.4. Biorthogonal wavelet

Biorthogonal wavelet families are upright and symmetrical. Such main wavelets are done by spline method [24].

Wavelet Transform is a different transformation method developed to overcome the shortcomings of non-stationary signals of the Fourier transform [13]. This analysis method is less sensitive to noise and can be easily applied to non-stationary signals. Therefore, the interest of those engaged in signal processing has shifted from the frequency-based Fourier Transform to the scale-based Wavelet Transform. The wavelet statement was put forward by Alfred Haar in 1909 [25]. Over time, Jean Marlet and Y. Meyer and their colleagues developed this method and in 1988, Stephane Mallat made in significant contributions. Later, international researchers such as Ingrid Daubechies and Ronald Coifman developed the method and brought it to the present. Wavelet transform has recently become a widely used method for multi-resolution analysis of signatures and images, such as radar target recognition, communication, texture image classification, and many other inland feature extraction [26].

The biggest advantage of wavelet transforms is that these transformations have a variable window size that window-size is wide for low frequencies and narrow for high frequencies. This ensures an optimum time-frequency resolution over all frequency ranges [13–15]. Discrete wavelet transform is a widely used method for feature extraction in recent years, such as signal processing, biomedical signal processing, and communication [14, 15]. The Discrete Wavelet Transform (DWT) is the sum of the signal that is scaled over time and multiplied by the offset values of the wavelet over time. The DWT function can be used at the desired level for the image or signal [23]. The DWT lower band separation method allows to obtain the discriminative characteristics of the signal by analyzing the component of the high frequency in a narrow range and the component of the low frequency in a wider band interval. The mathematical expression of the sub band separation up to the fourth level using DWT is shown in Eq (1) [27].


$$I_{BI}\left(u,v\right)=DWT\left\{I\left(x,y\right),B\in \left\{CA,CD1,CD2,CD3\right\},1<I<4\right\}$$ (1)

**Fig 1:**
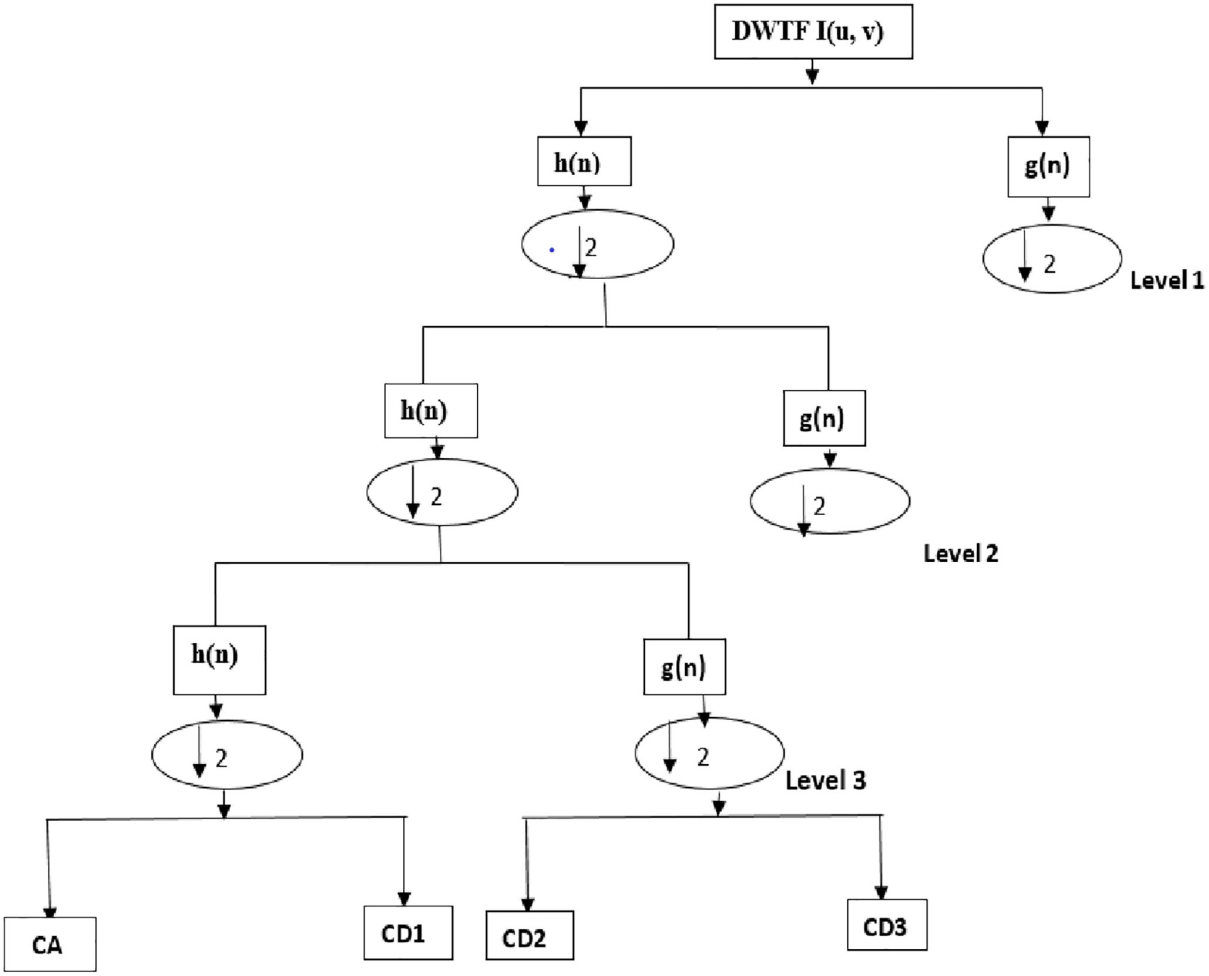
3 level DWT sub band separation of the original data signal.

Fig 1 shows the process of separating the mark to one level lower bands.

The g [n] in Fig 1 represents the high pass filter and the h [n] low pass filter. In the decomposition process for these sub-bands, h [n] expresses approximate coefficients for the operation, while g [n] expresses the detail coefficients for the operation [27].

Signaling is first subjected to filtering by line basis. As a result of this filtering, two groups of data are acquired without low and high pass filtering. Sample reduction is performed on the acquired data. These two subgroups are then again passed through low and high-pass filters according to the column principle. Two subgroups of data are acquired from these two subgroups. As a result, four sub data groups are acquired: horizontal, vertical and diagonal [14, 15, 27]. The approach data can be resumed in a similar way and DWT can be resumed. In Fig 1, CA approach, CD1 horizontal, CD2 vertical, CD3 diagonal is coefficient values.

## 2.2. Classification

### 2.2.1. Jensen Shannon Divergence (JSD)

JSD is one of the methods used to measure the distance between two probability distributions. In the literature, "information radius" or total divergence to average is also passed. By definition, the Kullback-Leibler Divergence can be thought of as a symmetrical state. That is, the distances from *t* to *u* for *t* and *u*, which are two probability distributions are not equal to every other at the distance of Kullback Leibler. The DKL (t || u)) distance which be from *t* to *u* with (DKL (t || u)) distance are not equal to every other [11]. However, JSD’s distance solves this problem and defines a symmetric distance function. The basic definition is as follows in Eq (2) [23, 28]:

$$JSD_{t,u}=\sum_{k}P\left(t\right)\;log(\frac{P\left(t\right)}{P\left(u\right)}+\;\left(1-P\left(t\right)\right)\;log(\frac{\left(1-P\left(t\right)\right)}{\left(1-P\left(u\right)\right)})$$ (2)

#### 2.2.2. Hellinger Divergence (HD)

*HD* is the distance function in the between *t* and *u*. The *HD* distance function [29] is shown in Eq (3).


$$HD_{t,u}=\sum_{k}(\sqrt{P\left(t\right)}\;\;-\sqrt{P(u}){)}^{2}$$ (3)

#### 2.2.3. Triangle Divergence (TD)

TD is distance function in the between t and u. The TD distance function [23, 28] is shown in Eq (4).

$$TD_{t,u}=\sum_{k}{\frac{\left|P\left(t\right)-P\left(u\right)\right|}{P\left(t\right)+P\left(u\right)}}^{2}$$ (4)

$$\;W_{JSDav,HDav,TDav}\left(p\right)=\sum_{r/p}\frac{\left(a_{pr}\right)}{K}JSD_{t,u},\;HD_{t,u},TD_{t,u}$$ (5)

$$W_{JSD,HD,TD}\left(p\right)=\frac{\;W_{JSDavg,HDavg,TDavg}\left(p\right)}{z\sum_{r/p}\;P\left(a_{pr}\right)\;\text{log}\;P\left(a_{pr}\right)}$$ (6)

where *W*_*JSDav*,*HDav*,*TDav*_ (*p*) are average weight calculation, *W*_*JSD*,*HD*,*TD*_ (*p*) are weight of the extracted *p* attribute for *JSD*, *HD* and *TD*, respectively. Total of cell numbers is K, z is standardization fixed, *a*_*pr*_ is *r*-th value in *p*-th feature, the goal attribute is *k*, *P*(*t*) is probabilistic value of the cells, *nSEM+nAFM*, *bSEM+bAFM* and *mSEM+mAFM*.

#### 2.2.4. Classification with DWTF-WJSD, DWT-WHD, and DWTF-WTD algorithms

These weights are written in the following in Eq (7), after calculating the weight for the cells [23, 30].

$$\left({\left(d_{JSD+HD+TD}\right)}_{SEM\;and\;AFM}\right)=argmax\;P\left(c\right)\prod_{a_{ij}\in d}\;P{\left(t\right)}^{W_{JSD,\;HD,TD}\left(i\right)}$$ (7)

where the goal attribute is *c*, *d*_*JSD*_ is test value of the JSD., *d*_*HD*_ is test value of the HD, *d*_*TD*_ is test value of the TD.

#### 2.2.5. Average of the DWTF-WJSD, DWT-WHD, and DWTF-WTD algorithms

Average Classification_SEM and AFM_ is average of the DWTF-WJSD, DWT-WHD, and DWTF-WTD Algorithms in Eq (8).

$$Average\;Classification_{SEM\;and\;AFM}=\frac{{\left(d_{JSD+HD+TD}\right)}_{SEM}+{\left(d_{JSD+HD+TD}\right)}_{AFM}}{3}$$ (8)

where (*d*_*JSD*_)_*SEM*_ is test value of the JSD for SEM images., (*d*_*HD*_)_*SEM*_ is test value of the HD for SEM images, (*d*_*TD*_)_*SEM*_ is test value of the TD for SEM, (*d*_*JSD*_)_*AFM*_ is test value of the JSD for AFM images., (*d*_*HD*_)_*AFM*_ is test value of the HD for AFM images, (*d*_*TD*_)_*AFM*_ is test value of the TD for AFM. *Average Classification*_*SEM and AFM*_ is average of the DWTF-WJSD, DWT-WHD, and DWTF-WTD Algorithms for SEM and AFM images.

## 3. Results

SEM cells in this article are nSEM, bSEM, and mSEM images. Also, AFM cells are nAFM, bAFM, and mAFM images. SEM cells are acquired from Sciece Faculty SEM Laboratuary in the Fırat University. Besides, AFM cells are acquired from Nanotechnology Laboratuary in the Fırat University. This article can be summarized as follows: Firstly, 45 SEM and AFM cervix images in the 512 × 512 size have been used for each total image type (nSEM, bSEM, mSEM nAFM, bAFM, and mAFM) obtained from Fırat University. The size of the cervical SEM and AFM images obtained from each of these images is 256 × 256. Therefore, 4x45 images were obtained from each type of image. So, total 540 SEM cells have been taken as 3x4x45 (nSEM, bSEM, and mSEM). Also, total 540 AFM cells have been taken as 3x4x45 (nAFM, bAFM, and mAFM).

The 2x270 images which taken from these 256 × 256 image regions obtained for each SEM and AFM (2 type) servix image were used in the training phase of DWTF-WJSD, DWTF-WHD, and DWTF-WTD algorithms. The remaining 2x270 images are used in the testing phase of these algorithms. Each of these 256 × 256 image parts obtained for each of the 45 cervix SEM and AFM images was applied Biorthogonal (bior), Symlet (sym), Coiflet (coif), and Daubechies (db) wavelet families. For this, the coif2, coif3, coif4, and coif5 which be types of the Coif wavelet family have been used for the SEM and AFM images. The sym2, sym3, sym4, and sym5 which be types of the sym wavelet family have been used for the SEM and AFM images. The bior1.3, bior2.8, bior1.5, and bior3.3 which be types of the bior wavelet family have been used for the SEM and AFM images. And, the DWT coefficients of these images are calculated according to Level 3.

**Fig 2:**
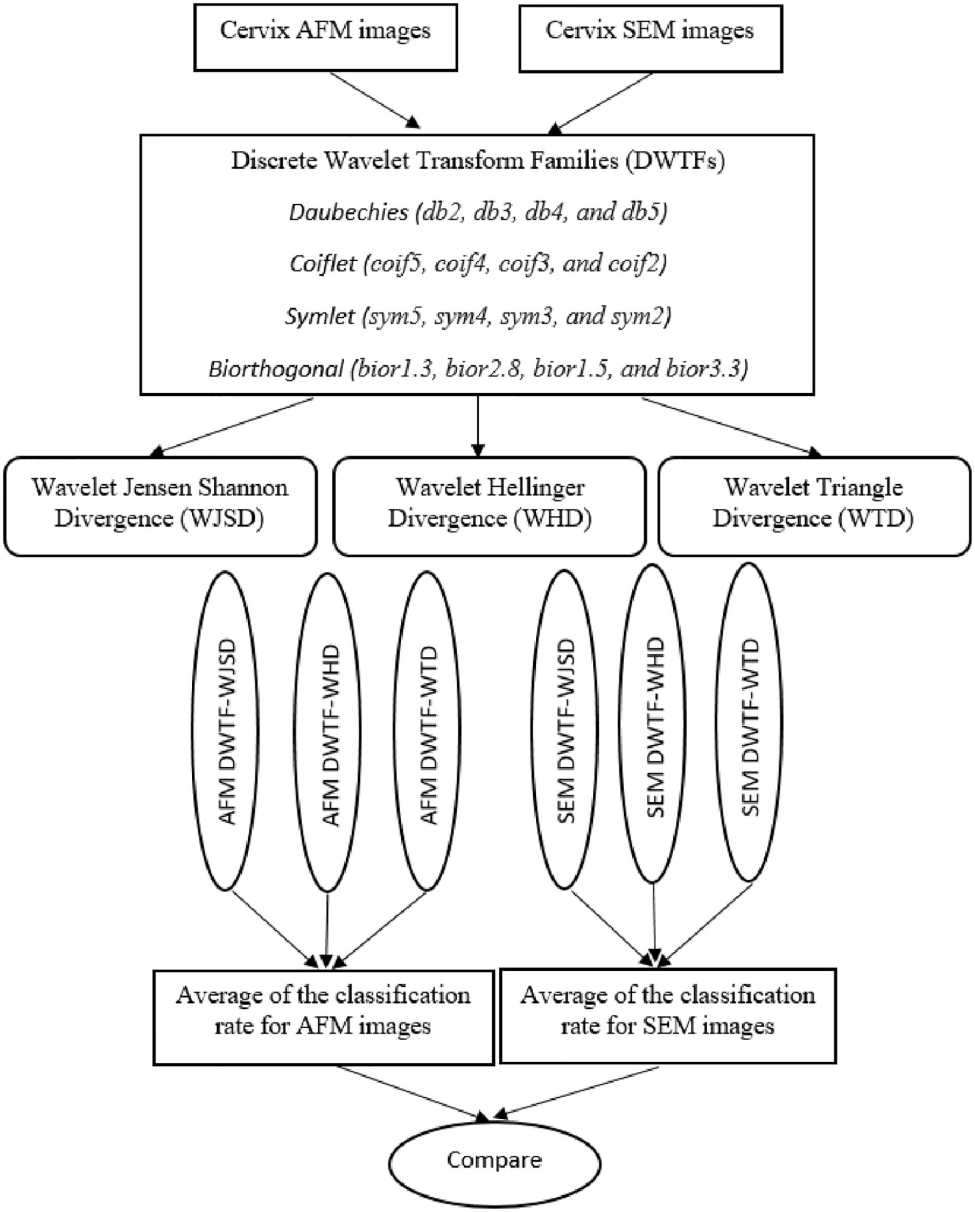
System based to DWTF-WJSD, DWTF-WHD, and DWTF-WTD algorithms for classification of the SEM and AFM cervix images.

Then, one approximate and three detail coefficients has been calculated with DWT method according to level 3 for each of the 256 × 256 size images. Homogeneity, contrast, angular second moment, entropy, mean, standard deviation, correlation, cluster prominence, dissimilarity, and cluster shade features have been calculated for each of the these approximate and detail coefficients. Thus, DWTFVs in the 4x10 size have been obtained for each of the 540 AFM and 540 SEM cervix images. The 270 pieces of these 540 and the remaining 270 pieces of them for both SEM cells and for AFM cells were used for training and testing steps of suggested algorithm in this study separately. The structure of DWTF-WJSD, DWTF-WHD, and DWTF-WTD algorithms is shown in Fig 2. This structure is used for classification of cervical SEM and AFM images.

Accuracy of classification results using DWTF-WJSD, DWTF-WHD, and DWTF-WTD algorithms in the Fig 2 has been tested. For this aim, 45 SEM and 45 AFM cervix images in the 512 × 512 size in this study have been used for type of the each image (nSEM, bSEM, mSEM nAFM, bAFM, and mAFM). The size of the SEM and AFM cervix images obtained from each of these images is 256 × 256. Therefore, 4x45 images were obtained from each type of image. So, total 540 SEM cells have been taken as 3x4x45 (nSEM, bSEM, and mSEM). Also, total 540 AFM cells have been taken as 3x4x45 (nAFM, bAFM, and mAFM). The 2x270 images which taken from these 256 × 256 image regions obtained for each SEM and AFM (2 type) servix image were used in the training phase of DWTF-WJSD, DWTF-WHD, and DWTF-WTD algorithms. The remaining 2x270 images are used in the testing phase of these algorithms.

## 4. Discussions

**Fig 3:**
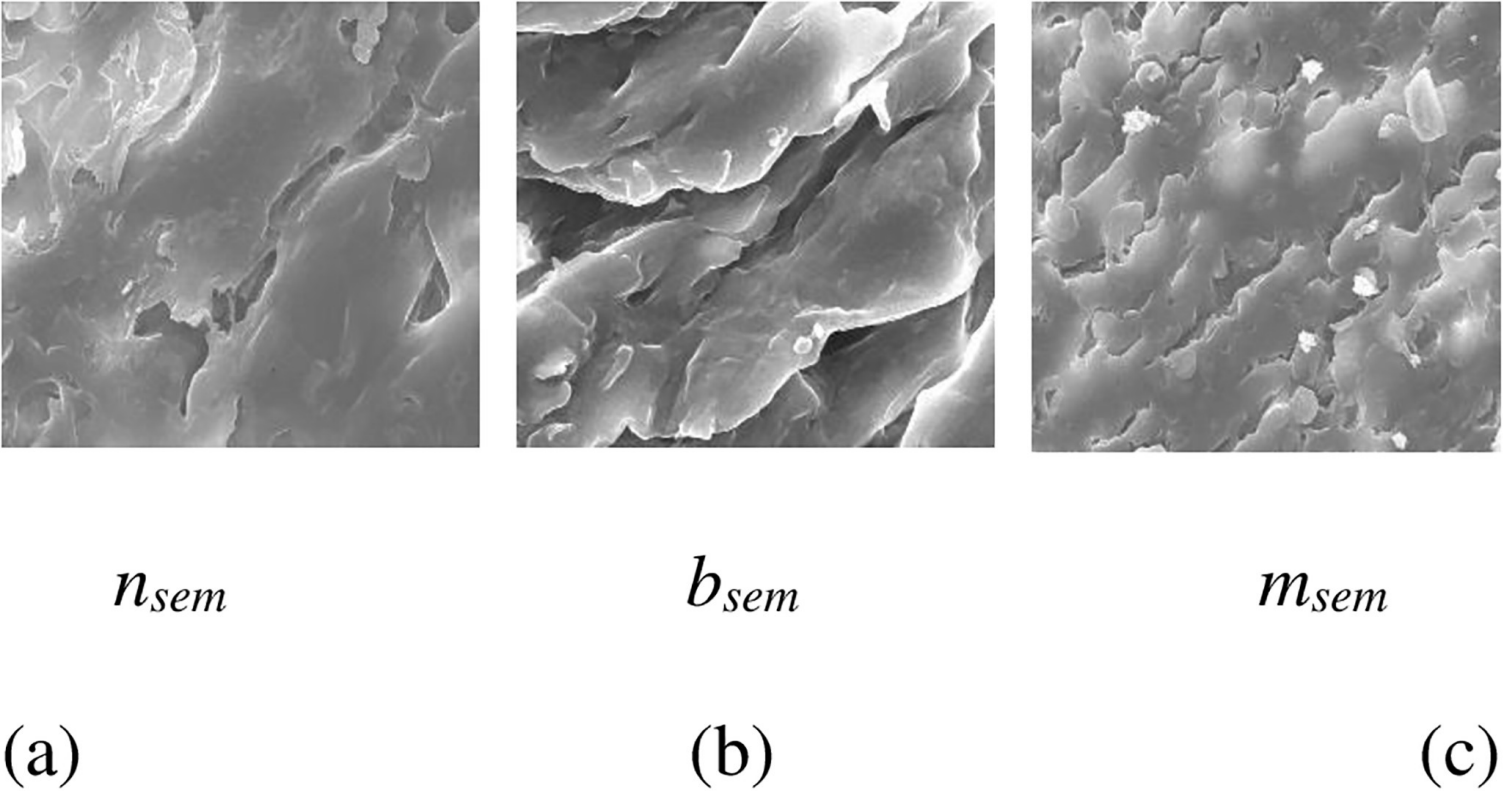
SEM cervix cell images. (a) nSEM (b) bSEM (c) mSEM.

SEM cells in this article are nSEM, bSEM, and mSEM images. Also, AFM cells are nAFM, bAFM, and mAFM images. SEM cells are acquired from Sciece Faculty SEM Laboratory in the Fırat University. Besides, AFM cells are acquired from Nanotechnology Laboratory in the Fırat University. nSEM is seen in Fig 3(a). bSEM is seen in Fig 3(b). mSEM is seen in Fig 3(c).

**Fig 4:**
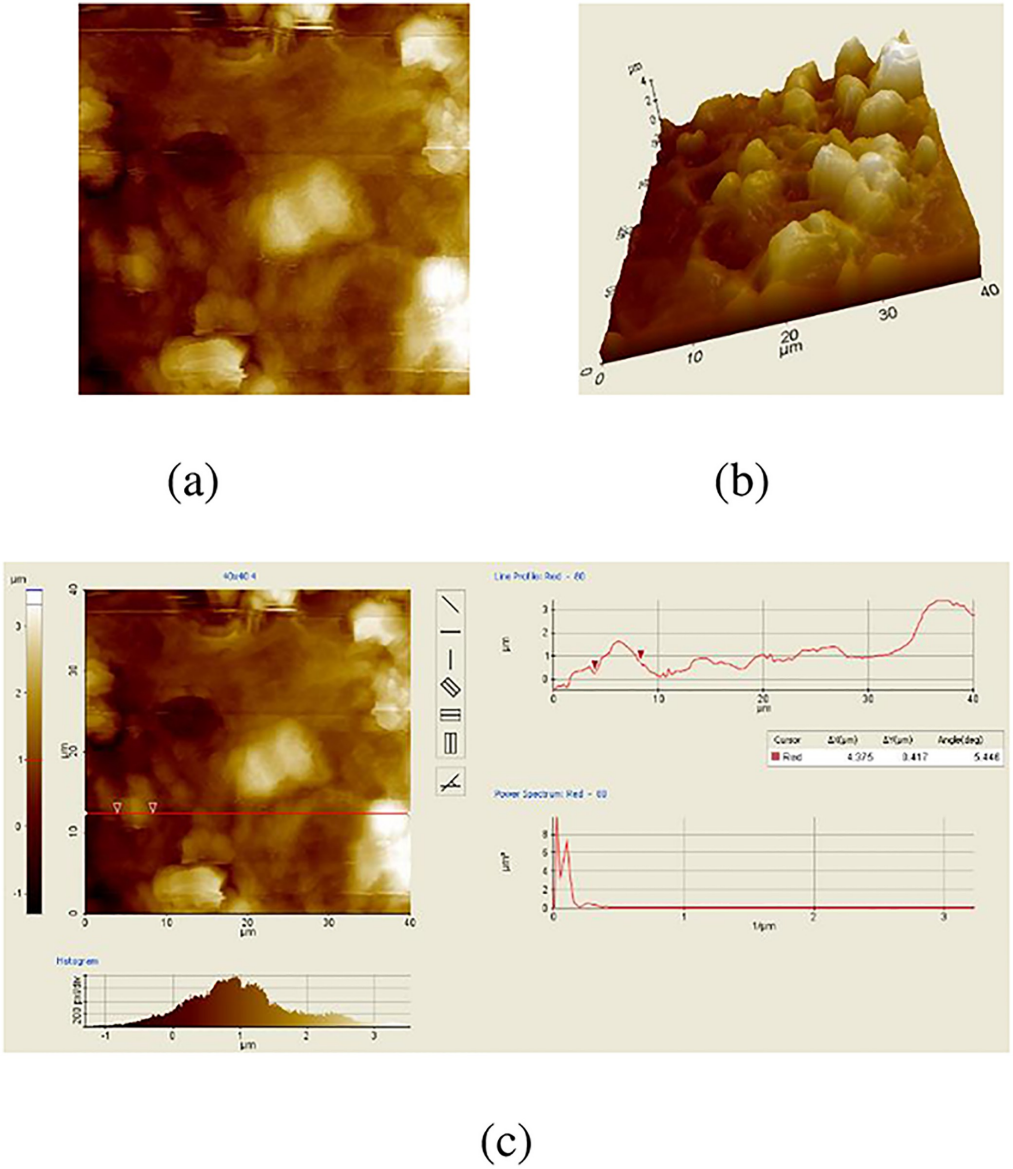
nAFM images. (a) Two-Dimensional nAFM cervix image (b) Three-dimensional nAFM cervix image (c) Particle volume and surface roughness nAFM cervix image.

Fig 4(a) shows the nAFM cervix cell images. Fig 4(a) is an image the two dimensional. Three dimensional image of nAFM is seen in Fig 4(b). Particle volume and surface roughness values of the nAFM image are seen in Fig 4(c). Values the of surface roughness and particle volume for nAFM images are measured as 50.23 *nm* and 4.375 *μm*.

**Fig 5:**
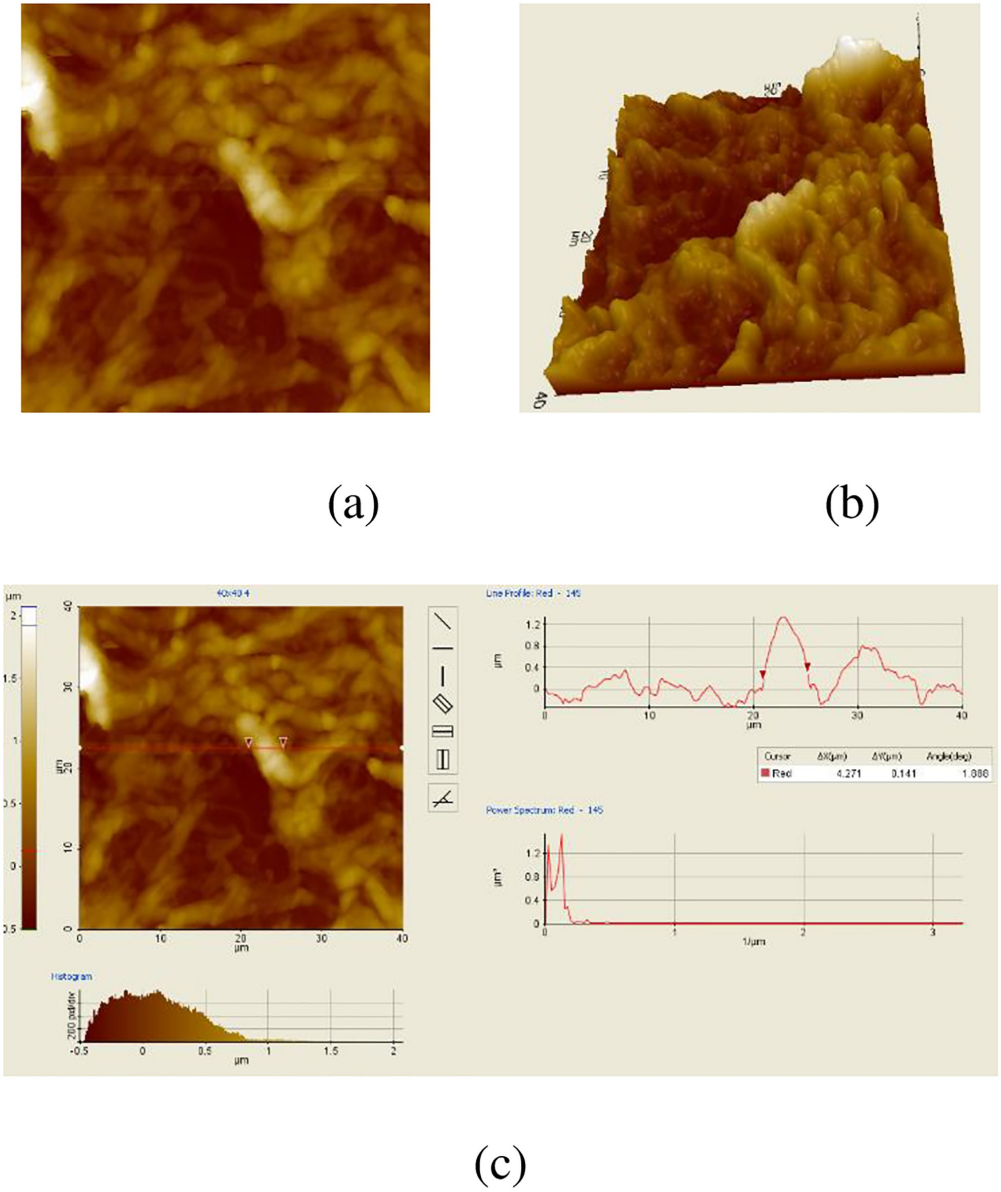
bAFM images. (a) Two-Dimensional bAFM cervix image (b) Three-dimensional bAFM cervix image (c) Particle volume and surface roughness bAFM cervix image.

Fig 5(a) shows the bAFM cervix cell images. Fig 5(a) is an image the two dimensional. Three dimensional image of bAFM is seen in Fig 5(b). Particle volume and surface roughness values of the bAFM image are seen in Fig 5(c). Values the of surface roughness and particle volume for bAFM images are measured as 244.654 *nm* and 4.271 *μm*.

**Fig 6:**
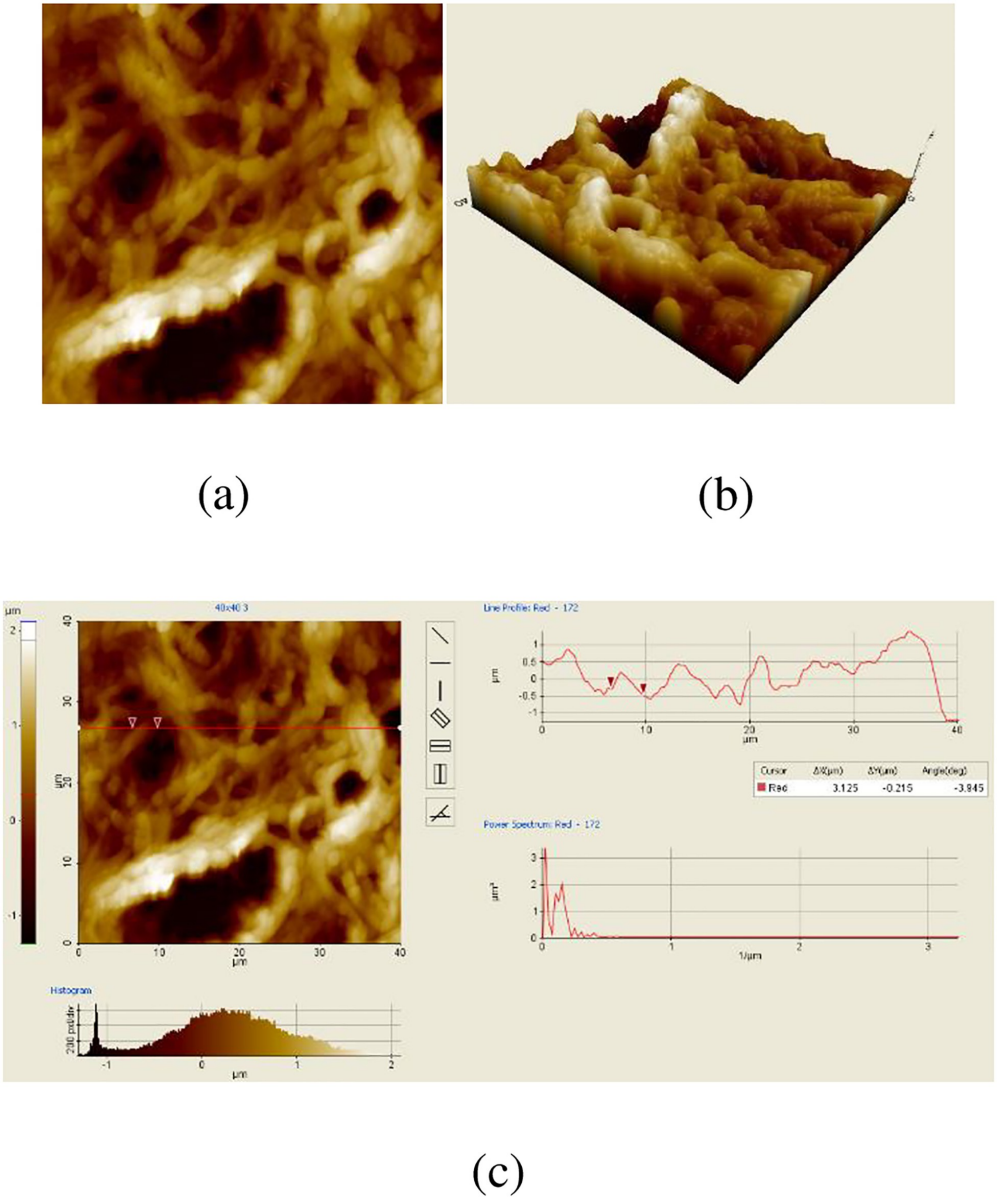
mAFM images. (a) Two-Dimensional mAFM cervix image (b) Three-dimensional mAFM cervix image (c) Particle volume and surface roughness mAFM cervix image.

Fig 6(a) shows the mAFM cervix cell images. Fig 6(a) is an image the two dimensional. Three dimensional image of mAFM is seen in Fig 6(b). Particle volume and surface roughness values of the nAFM image are seen in Fig 6(c). Values the of surface roughness and particle volume for mAFM images are measured as 456.145 *nm* and 3.125 *μm*.

**Fig 7:**
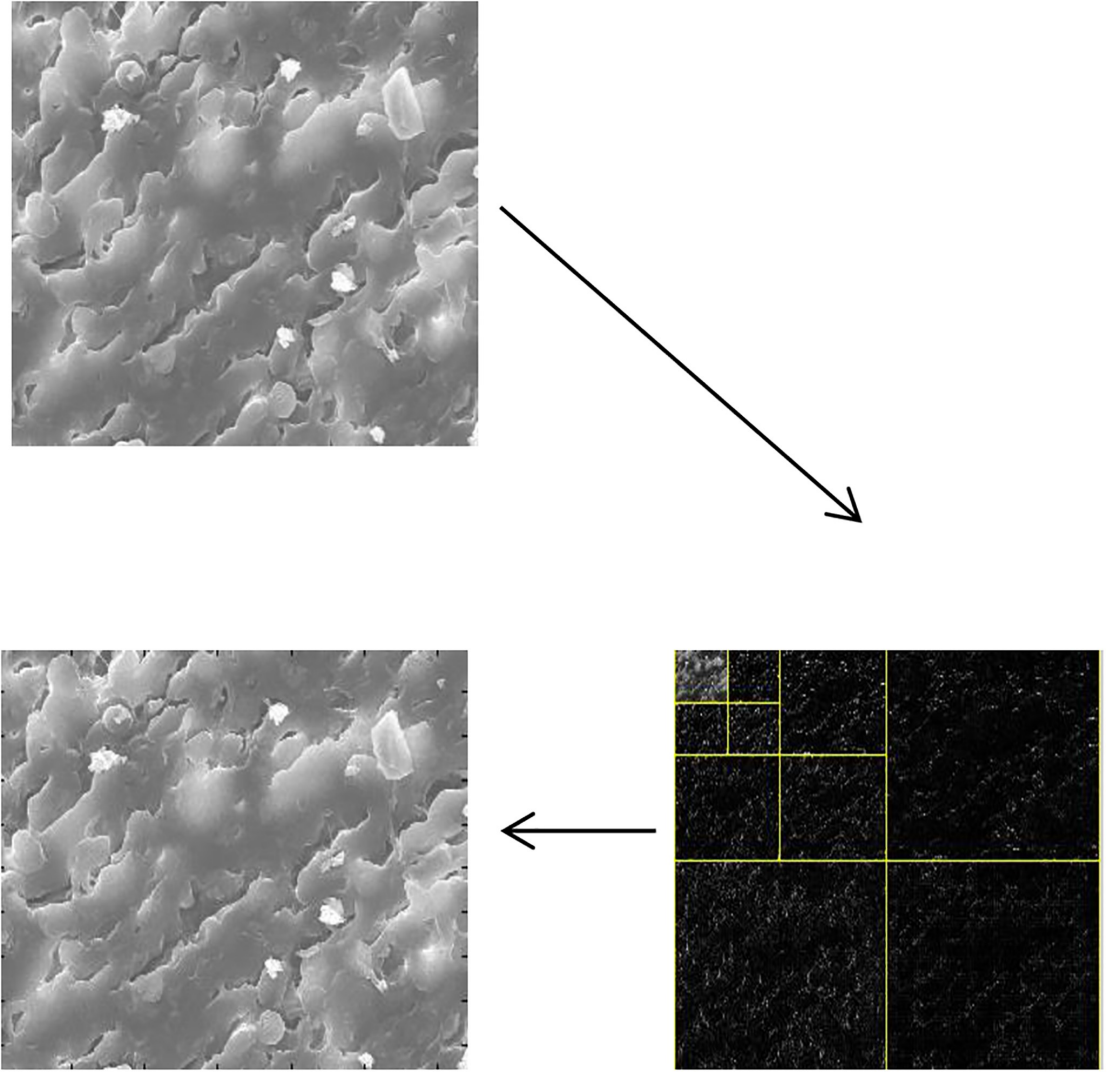
mSEM cervix image at level 3 which used sym3 DWTFV.

Fig 7 represents the mSEM cervix image at level 3 which used sym3 DWTFV.

**Fig 8:**
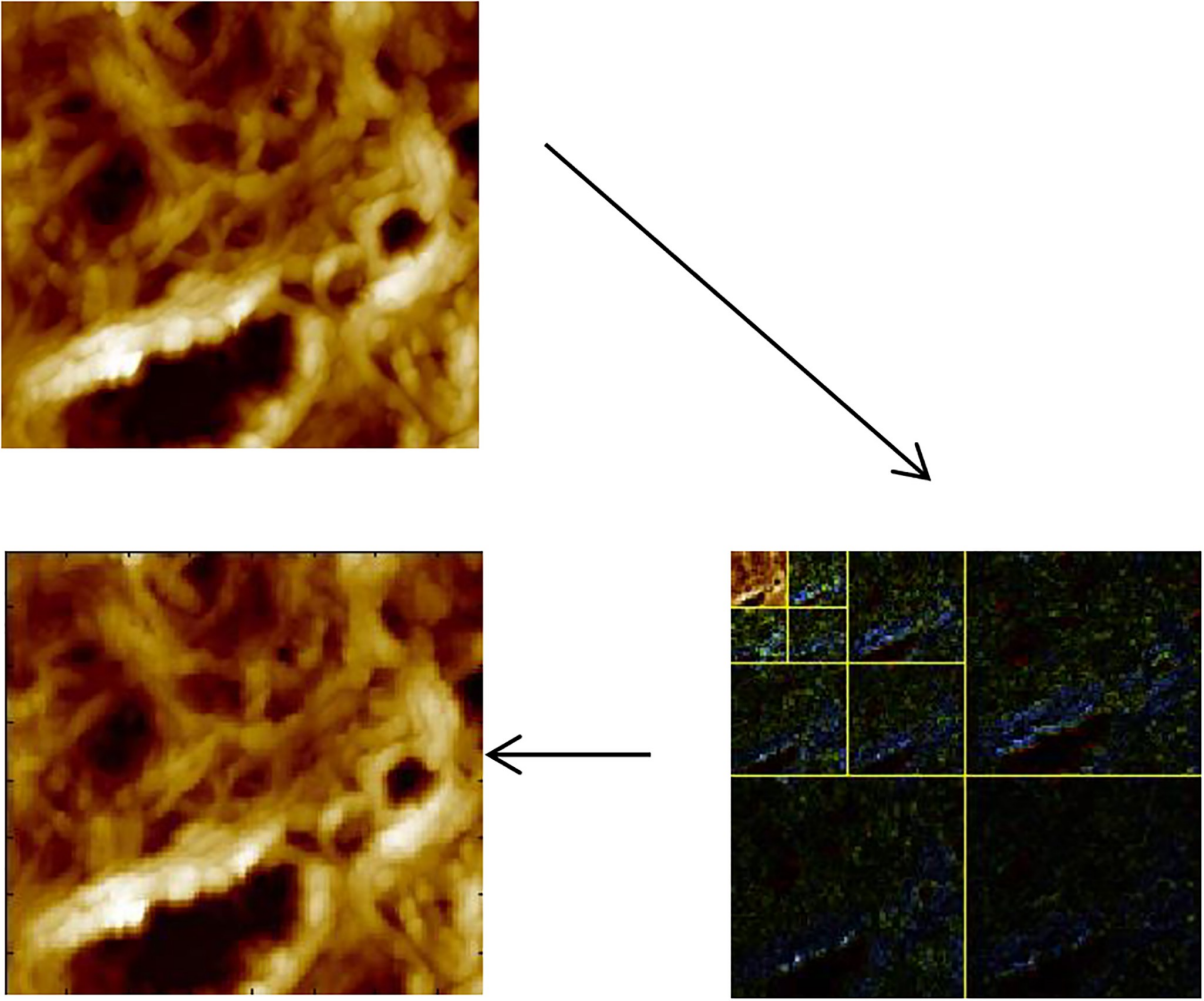
mAFM cervix image at level 3 which used sym2 DWTFV.

Fig 8 represents the mAFM cervix image at level 3 which used sym2 DWTFV.

45 SEM and 45 AFM cervix images in the 512 × 512 size in this study have been used for type of the each image (nSEM, bSEM, mSEM nAFM, bAFM, and mAFM) obtained from Fırat University. The size of the cervical SEM and AFM images obtained from each of these images is 256 × 256. Therefore, 4x45 images were obtained from each type of image. So, total 540 SEM cells have been taken as 3x4x45 (nSEM, bSEM, and mSEM). Also, total 540 AFM cells have been taken as 3x4x45 (nAFM, bAFM, and mAFM). The 2x270 images which taken from these 256 × 256 image regions obtained for each SEM and AFM (2 type) servix image were used in the training phase of DWTF-WJSD, DWTF-WHD, and DWTF-WTD algorithms. The remaining 2x270 images are used in the testing phase of these algorithms. The performance of this Cervical SEM and AFM image classification obtained using the DWTF-WJSD, DWTF-WHD, and DWTF-WTD algorithms is given in Tables 1 and 2. Average the classification rate for DWTFVs with DWTF-WJSD algorithm for AFM images in Table 1 is obtained as 98.35%. average the classification rate for DWTFVs with DWTF-WHD algorithm for AFM images is obtained as 98.09%. Average the classification rate for DWTFVs with DWTF-WTD algorithm for AFM images is obtained as 98.45%. Average of these averages founded from DWTF-WJSD, DWTF-WHD, and DWTF-WTD algorithms are obtained as 98.29%.

**Table 1:** The classification rates of DWTF-WJSD, DWTF-WHD, and DWTF-WTD algorithms used for AFM images.

AFM Cervix Cells	Classification Rate for AFM images (%)															
	DWTFV for AFM images															
	db2	db3	db4	db5	bior1.5	bior1.3	bior2.8	bior3.3	coif2	coif3	coif4	coif5	sym2	sym3	sym4	sym5
nAFM	98.66	97.88	98.18	97.00	97.18	97.00	98.88	98.88	96.18	97.88	99.50	99.88	98.44	98.18	96.00	97.55
bAFM	98.18	98.44	98.44	98.50	99.55	99.88	97.50	97.66	98.66	100	100	97.00	100	98.00	99.55	97.66
mAFM	99.88	99.00	98.62	99.50	98.66	97.66	98.00	97.66	96.50	99.50	96.50	97.66	99.55	100	97.44	99.00
DWTF-WJSD (%)	98.90	98.44	98.41	98.33	98.46	98.18	98.12	98.06	97.11	99.12	98.66	98.18	99.33	98.72	97.66	98.07
nAFM	100	99.66	97.66	99.00	98.44	99.62	98.66	96.62	98.50	96.44	97.18	98.50	100	98.44	96.62	98.44
bAFM	96.62	99.50	100	97.00	99.62	98.00	98.50	100	98.00	97.62	98.00	98.44	97.50	96.88	96.00	95.00
mAFM	98.00	98.00	99.18	98.50	96.44	97.00	99.00	96.66	99.18	98.50	98.66	99.00	98.62	98.50	96.88	99.18
DWTF-WHD (%)	98.20	99.05	98.94	98.16	98.16	98.20	98.72	97.76	98.56	97.52	97.94	98.64	97.70	97.94	96.5	97.54
nAFM	99.44	98.00	99.88	96.50	98.66	96.88	98.66	100	97.44	99.18	97.18	98.44	98.66	98.44	98.00	98.00
bAFM	97.50	99.44	98.18	98.44	99.44	99.44	96.50	97.88	96.50	98.50	99.50	98.18	98.50	98.18	100	98.50
mAFM	98.88	99.00	100	99.00	98.44	97.50	99.50	98.18	98.88	98.88	99.66	96.88	96.62	98.50	98.50	99.00
DWTF-WTD (%)	98.60	98.81	99.35	97.98	99.03	97.94	98.22	98.68	97.60	98.85	98.78	97.83	97.92	98.37	98.83	98.50

**Table 2:** The classification rates of DWTF-WJSD, DWTF-WHD, and DWTF-WTD algorithms used for SEM images.

SEM Cervix Cells	Classification Rate for SEM images (%)															
	DWTFV for SEM images															
	db2	db3	db4	db5	bior1.5	bior1.3	bior2.8	bior3.3	coif2	coif3	coif4	coif5	sym2	sym3	sym4	sym5
nSEM	97.50	97.77	96.00	97.44	96.22	96.12	97.77	97.88	95.88	99.12	97.50	97.50	97.12	97.77	96.50	95.88
bSEM	98.00	97.22	96.12	96.33	98.00	97.44	95.88	96.12	98.00	100	97.00	96.88	95.55	97.44	98.00	98.88
mSEM	97.22	96.55	98.24	97.50	97.12	96.88	96.50	98.50	96.88	95.22	97.22	96.50	96.88	96.88	97.22	97.44
DWTF-WJSD (%)	97.57	97.18	96.78	97.09	97.11	96.81	96.71	97.50	96.92	98.11	97.24	96.96	96.51	97.36	97.24	97.40
nSEM	96.77	96.55	96.44	99.12	95.22	97.88	96.12	95.50	96.12	99.88	96.50	97.50	96.33	98.00	96.50	96.44
bSEM	95.22	95.00	96.22	96.22	99.88	97.00	97.88	97.22	97.22	96.22	97.88	96.00	95.77	96.22	97.00	97.88
mSEM	98.12	98.24	98.88	96.88	97.44	95.88	97.77	99.00	95.50	97.44	95.88	97.12	99.44	97.55	98.55	96.88
DWTF-WHD (%)	96.70	96.59	97.18	97.40	97.51	96.92	97.25	97.24	96.28	97.84	96.75	96.87	97.18	97.25	97.35	97.06
nSEM	97.88	95.88	97.44	98.44	95.44	95.00	96.50	96.00	96.50	96.33	96.88	97.12	98.44	96.77	97.88	96.44
bSEM	97.22	96.12	97.12	96.50	99.50	98.88	97.44	97.50	97.33	99.44	97.50	97.88	97.12	100	96.44	97.18
mSEM	97.44	97.88	96.88	95.50	97.00	96.50	95.88	97.12	97.44	97.24	97.18	96.44	95.88	95.88	97.55	97.00
DWTF-WTD (%)	97.51	96.62	97.14	96.81	97.31	96.79	96.60	96.87	97.09	97.67	97.18	97.14	97.14	97.55	97.29	96.87

Average the classification rate for DWTFVs with DWTF-WJSD algorithm for SEM images in Table 2 is obtained as 97.15%. average the classification rate for DWTFVs with DWTF-WHD algorithm for SEM images is obtained as 97.08%. Average the classification rate for DWTFVs with DWTF-WTD algorithm for SEM images is obtained as 97.09%. Average of these averages founded from DWTF-WJSD, DWTF-WHD, and DWTF-WTD algorithms are obtained as 97.10%

## 5. Conclusion

To date, there are different studies in the field of machine learning in different types of cancer [28–30]. However, to date, many studies have been carried out in the field of biomedicine and different types of cancer [31–36]. However, to date, there is no other study using AFM and SEM images together for early cervical cancer diagnosis. SEM cells in this article are nSEM, bSEM, and mSEM images. Also, AFM cells are nAFM, bAFM, and mAFM images. SEM cells are acquired from Sciece Faculty SEM Laboratory in the Fırat University. Besides, AFM cells are acquired from Nanotechnology Laboratory in the Fırat University. 270 AFM and 270 SEM images were used for training purposes. 270 AFM and 270 SEM images were used for testing purposes. Classification rates of the nAFM, bAFM, and mAFM cervix images are shown in Table 1.

Similarly, Classification rates of the nSEM, bSEM, and mSEM cervix images are shown in Table 2.

**Fig 9:**
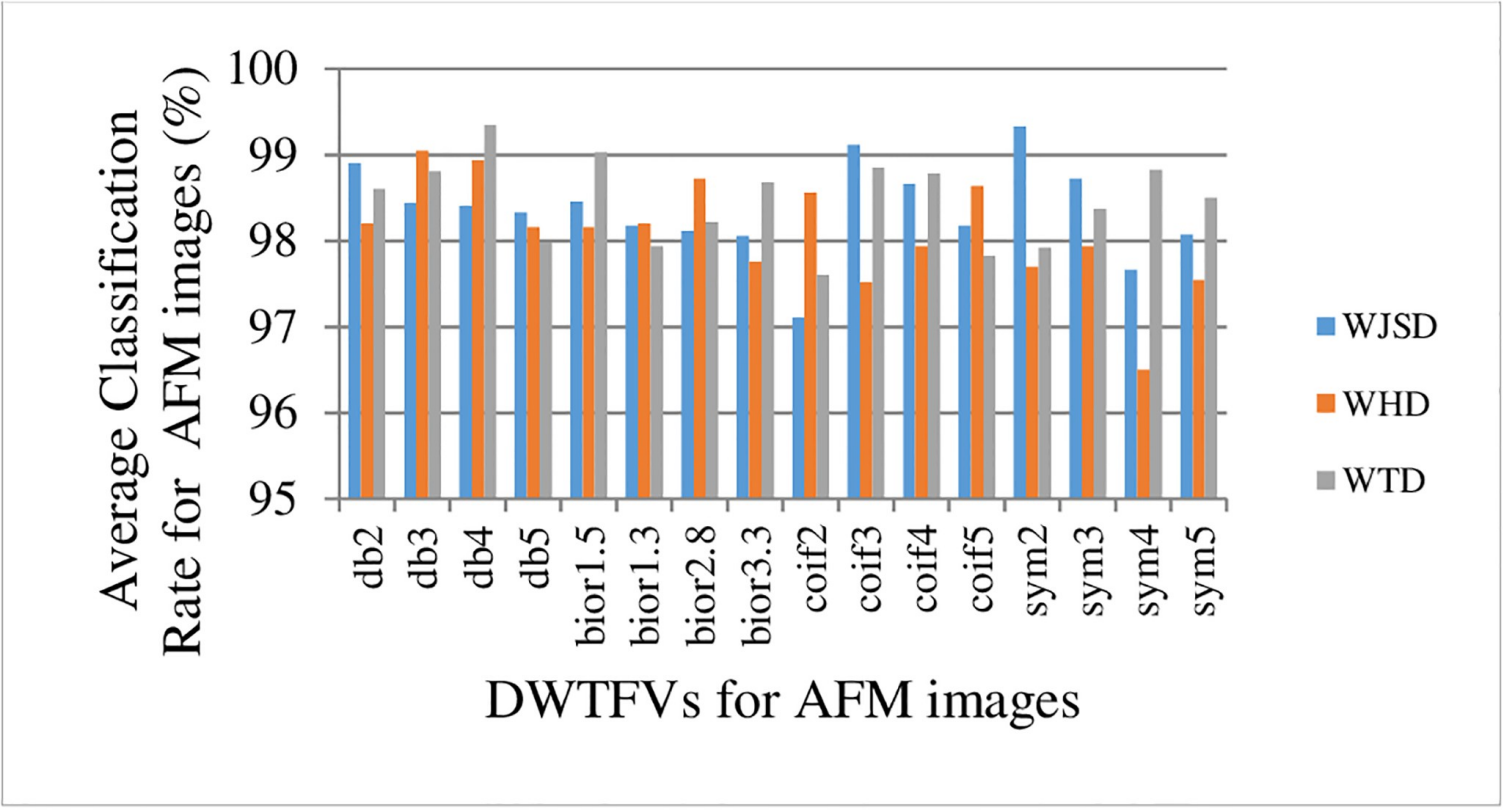
Average classification rates (%) for AFM images of the DWTF-WJSD, DWTF-WHD, and DWTF-WTD algorithms.

These AFM classification rates found in Fig 9 are obtained from DWTF-WJSD, DWTF-WHD, and DWTF-WTD algorithms.

**Fig 10:**
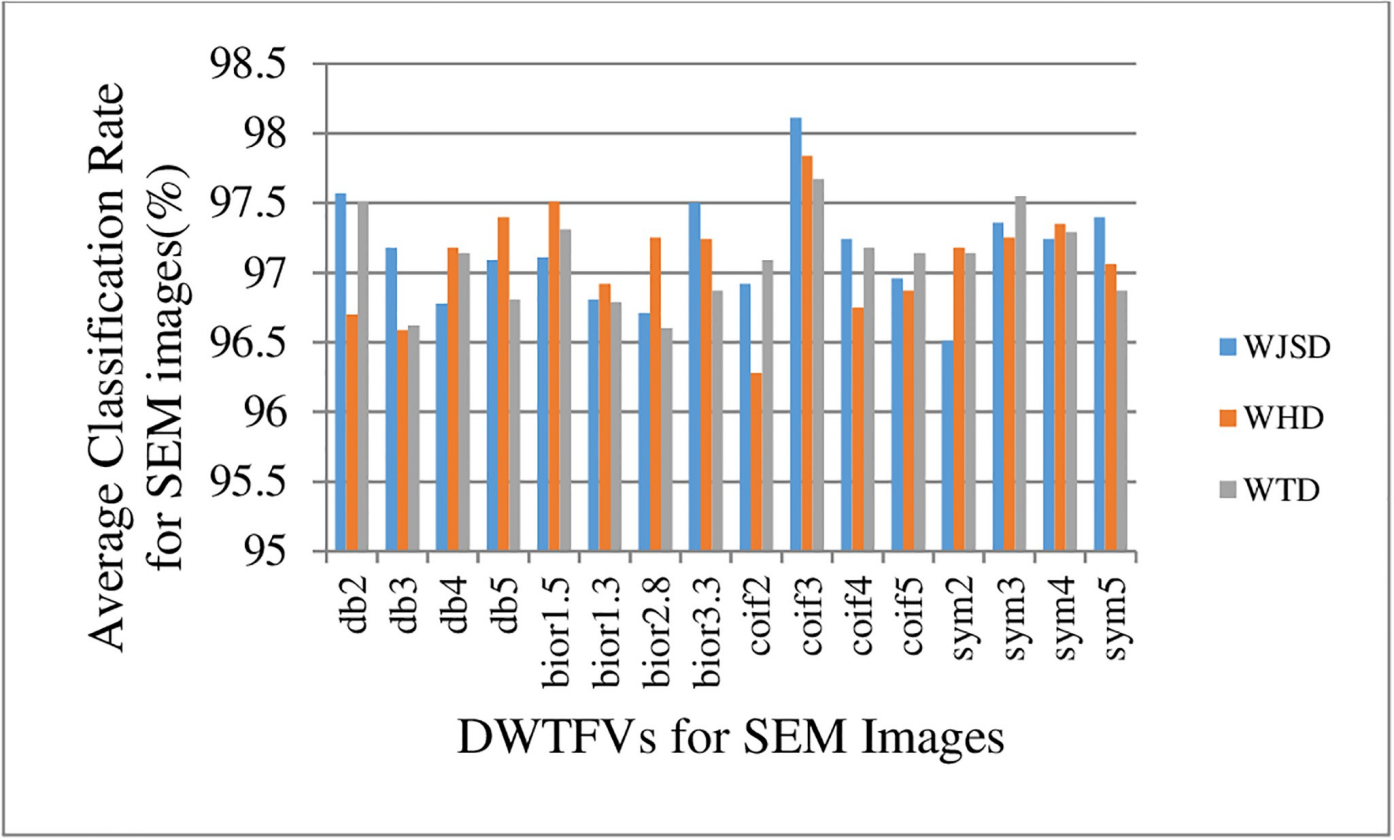
Average classification rates (%) for SEM images of the DWTF-WJSD, DWTF-WHD, and DWTF-WTD algorithms.

These SEM classification rates found in Fig 10 are obtained from DWTF-WJSD, DWTF-WHD, and DWTF-WTD algorithms.

**Fig 11:**
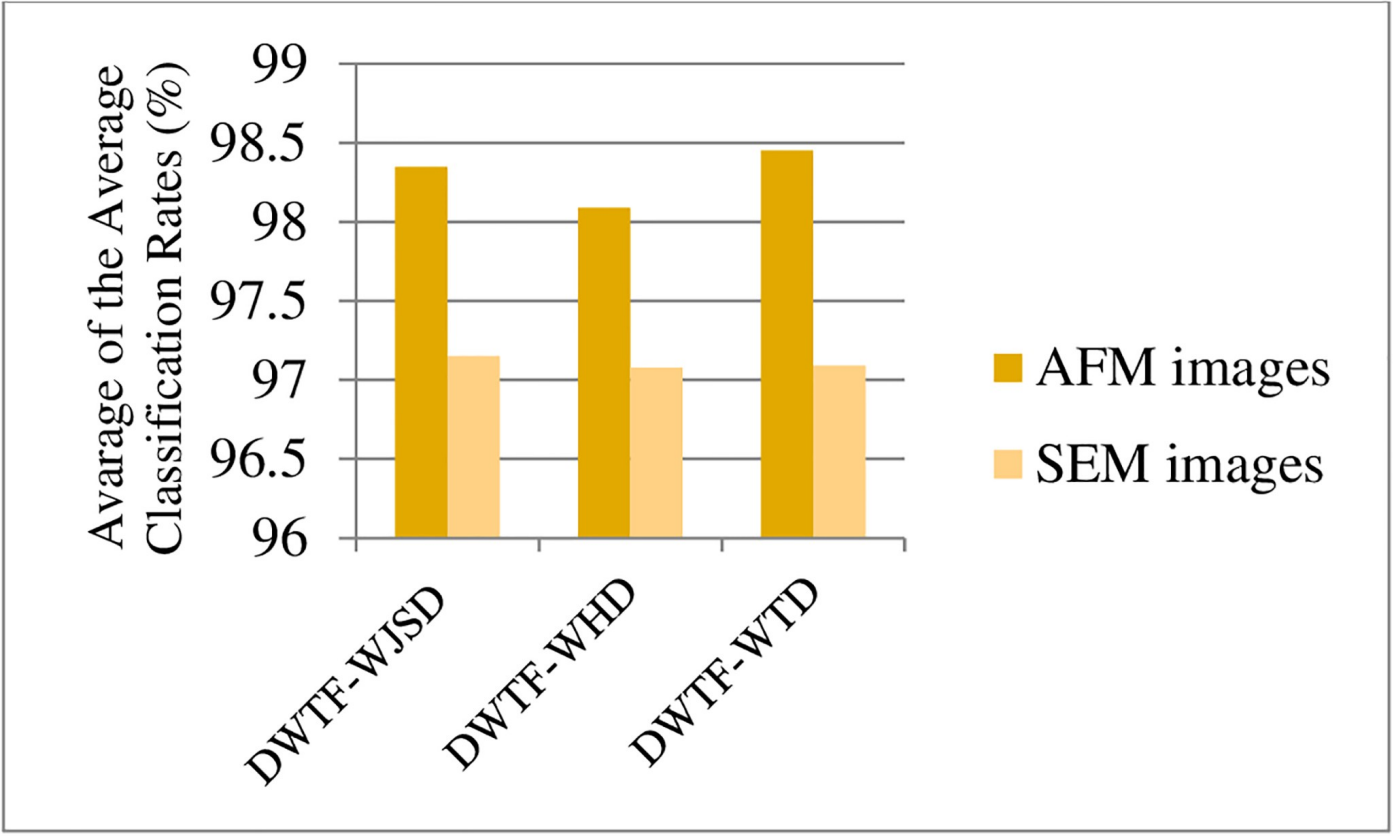
Average of classification rate (%) obtained with DWTF-WJSD, DWTF-WHD, and DWTF-WTD algorithms.

Average the classification rate for DWTFVs with DWTF-WJSD algorithm is obtained as 98.35% for AFM images in Fig 11.

Average the classification rate for DWTFVs with DWTF-WHD algorithm is obtained as 98.09% for AFM images. Average the classification rate for DWTFVs with DWTF-WTD algorithm is obtained as 98.45% for AFM images. Average of these averages founded from DWTF-WJSD, DWTF-WHD, and DWTF-WTD algorithms are obtained as 98.29%. Average the classification rate for DWTFVs with DWTF-WJSD algorithm is obtained as 97.15% for SEM images in Fig 11. Average the classification rate for DWTFVs with DWTF-WHD algorithm is obtained as 97.08% for SEM images. Average the classification rate for DWTFVs is obtained as 97.09% with DWTF-WTD algorithm for SEM images. Average of these averages founded from DWTF-WJSD, DWTF-WHD, and DWTF-WTD algorithms are obtained as 97.10%.

**Fig 12:**
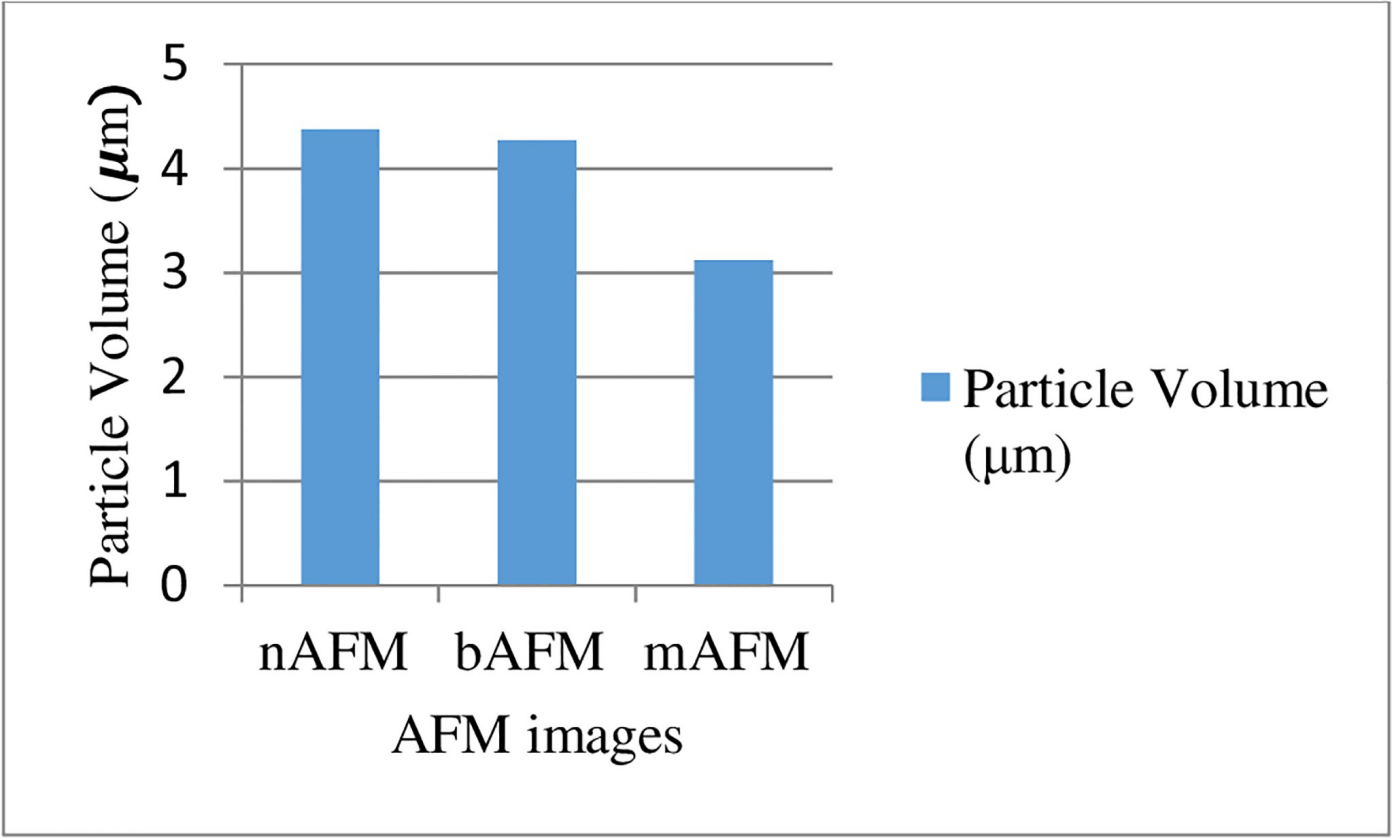
Particle volume (micrometer (*μm*)) values of nAFM, bAFM, and mAFM.

4.375 *μm*, 4.271 *μm* and 3.125 *μm* seen in Fig 12 are the particle volume values of the nAFM, bAFM, and mAFM images, respectively. Also, mAFM particle volume value is smaller than nAFM and bAFM particle volume value.

**Fig 13:**
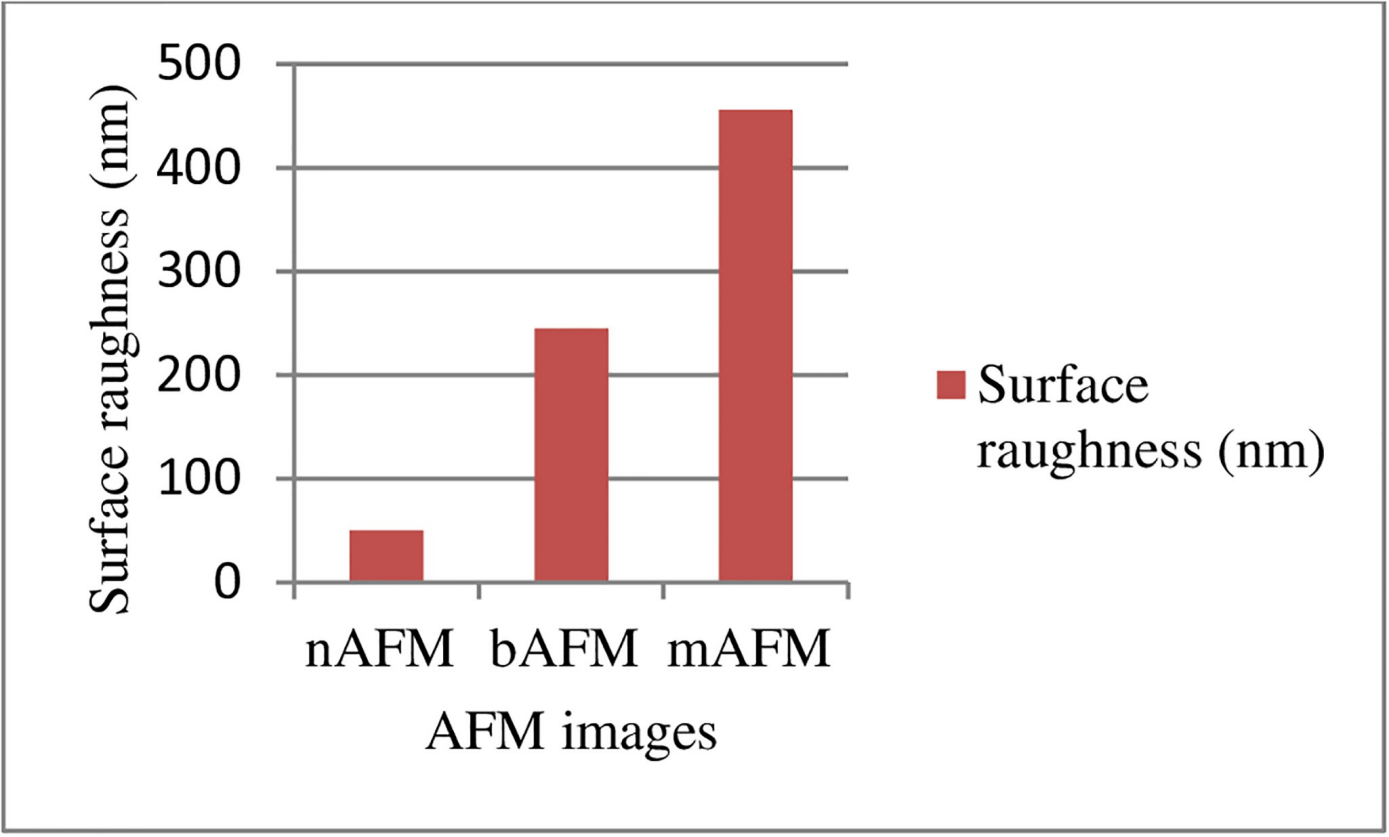
Surface roughness (nanometer (*nm*)) values of nAFM, bAFM, and mAFM images.

50.23 *nm*, 244.654 *nm* and 456.145 *nm* seen in Fig 13 are the surface roughness values of the nAFM, bAFM, and mAFM images, respectively. Also, mAFM surface roughness value is smaller than nAFM and bAFM surface roughness value.

The fact that it has a fast and expensive structure is one of the advantages of DWTF-WJSD, DWTF-WHD, and DWTF-WTD algorithms. This system can be used to classify images of ultrasonographic, mammographic, endoscopic and different types of cancer in subsequent studies.

## Supporting information

S1 File (DOCX)
