# Supplementary material for: Photodiagnosis and photodynamic recognition of cervical cancer with SEM and AFM images
Source: PLoS One. 2025 Feb 6;20(2):e0316544. doi: 10.1371/journal.pone.0316544 (PMC11801595; doi:10.1371/journal.pone.0316544)
Supplement: S1 File — (DOCX) [file pone.0316544.s001.docx]

The study adhered to the Declaration of Helsinki and was approved by the local ethics review board. In this study, we used real medical images in order to evaluate the cervical cancer diagnosis. We sought Non-Interventional Clinical Researches Ethics Board in Firat University in order to obtain patient records coming from Elazig and its neighbor cities in Turkey. The medical image usage was approved with decision number of 07–05 by the related board. After this, we collected the patients’ medical records belonging to Firat University Hospital. Patients’ medical records were taken suitably to the ethics rules of the hospital. Medical tissue samples are determined from Firat University Medicine Faculty Pathology Laboratory. Our study is a retrospective study. Ethical approval was obtained before starting the study. It was conducted in accordance with the Declaration of Helsinki. Our relevant ethics committee approval has been uploaded to the other files sections. Our study started on 01/05/2016 and ended on 01/05/2021. Our study is a retrospective study. The data used in the study were obtained from medical archive records after ethical approval. Our ethics committee therefore did not require a consent form and waived the need for consent. Therefore, consent was not obtained. No patient data under the age of 18 was used in the medical data used in the study. The medical records required for our study were accessed between 01/05/2016 and 01/05/2021. All data were obtained from archived medical records after obtaining relevant permissions and approvals. No extra intervention was given to the participants.
